# Supplementary material for: The economic impact of premature mortality in Cabo Verde: 2016–2020
Source: PLoS One. 2023 May 24;18(5):e0278590. doi: 10.1371/journal.pone.0278590 (PMC10208520; doi:10.1371/journal.pone.0278590)
Supplement: S1 Appendix — (DOCX) [file pone.0278590.s001.docx]

**Appendix 1: Characterization of the population, deaths from all causes, 2016 to 2020**

| **Variables** | | **n** | **%** |
| --- | --- | --- | --- |
| Sex | Male | 7807 | 54.9 |
|  | Female | 6401 | 45.1 |
|  | **Total** | **14208** | **100** |
| Age group | 0 to 4 years | 1405 | 9.9 |
|  | 5 to 9 years | 47 | 0.3 |
|  | 10 to 14 years | 62 | 0.4 |
|  | 15 to 19 years | 103 | 0.7 |
|  | 20 to 24 years | 200 | 1.4 |
|  | 25 to 29 years | 235 | 1.7 |
|  | 30 to 34 years | 296 | 2.1 |
|  | 35 to 39 years | 424 | 3.0 |
|  | 40 to 44 years | 472 | 3.3 |
|  | 45 to 49 years | 550 | 3.9 |
|  | 50 to 54 years | 714 | 5.0 |
|  | 55 to 59 years | 842 | 5.9 |
|  | 60 to 64 years | 798 | 5.6 |
|  | 65 to 69 years | 699 | 4.9 |
|  | 70 to 74 years | 644 | 4.5 |
|  | 75 to 79 years | 992 | 7.0 |
|  | 80 to 84 years | 1723 | 12.1 |
|  | 85 and over | 4003 | 28.2 |
|  | **Total** | **14209** | **100.0** |
| Marital status | single | 6815 | 56.2 |
|  | Married / de facto union | 3563 | 29.4 |
|  | Divorced / Separated | 192 | 1.6 |
|  | Widower | 1561 | 12.9 |
|  | Other | 6 | 0.05 |
|  | **Total** | **12137** | **100** |
| Municipality | Ribeira Grande | 697 | 4.9 |
|  | Paul | 254 | 1.8 |
|  | Porto Novo | 508 | 3.6 |
|  | São Vincent | 2543 | 17.9 |
|  | Ribeira Brava | 335 | 2.4 |
|  | Tarrafal de São Nicolau | 187 | 1.3 |
|  | Sal | 569 | 4.0 |
|  | Boavista | 262 | 1.8 |
|  | Maio | 204 | 1.4 |
|  | Tarrafal | 568 | 4 |
|  | Santa Catarina | 1326 | 9.3 |
|  | Santa Cruz | 688 | 4.8 |
|  | Praia | 3274 | 23 |
|  | São Domingos | 399 | 2.8 |
|  | São Miguel | 426 | 3.0 |
|  | São Salvador do Mundo | 246 | 1.7 |
|  | São Lourenço dos Órgãos | 205 | 1.4 |
|  | Ribeira Grande de Santiago | 196 | 1.4 |
|  | Mosteiros | 235 | 1.7 |
|  | São Felipe | 646 | 4.5 |
|  | Santa Catarina do Fogo | 121 | 0.9 |
|  | Brava | 215 | 1.5 |
|  | Others | 105 | 0.7 |
|  | **Total** | **14209** | **100** |
