# Supplementary material for: The economic impact of premature mortality in Cabo Verde: 2016–2020
Source: PLoS One. 2023 May 24;18(5):e0278590. doi: 10.1371/journal.pone.0278590 (PMC10208520; doi:10.1371/journal.pone.0278590)
Supplement: S2 Appendix — (DOCX) [file pone.0278590.s002.docx]

**Appendix 2: Number of deaths by causes, Cape Verde, 2016 to 2020.**

| **Cause of death** | **n** | **%** |
| --- | --- | --- |
| Diseases of the circulatory system | 4180 | 29.4 |
| Diseases of the respiratory system | 1838 | 12.9 |
| Neoplasms [tumors] | 1694 | 11.9 |
| Certain infectious and parasitic diseases | 1516 | 10.7 |
| Symptoms, signs and abnormal clinical and laboratory findings, not elsewhere classified | 1164 | 8.2 |
| Certain conditions originating in the perinatal period | 954 | 6.7 |
| External causes of morbidity and mortality | 600 | 4.2 |
| Injury, poisoning and certain other consequences of external causes | 572 | 4.0 |
| Diseases of the digestive system | 451 | 3.2 |
| Endocrine, nutritional and metabolic diseases | 419 | 2.9 |
| Diseases of the genitourinary system | 243 | 1.7 |
| Mental and behavioral disorders | 179 | 1.3 |
| Diseases of the nervous system | 169 | 1.2 |
| Congenital malformations, deformations and chromosomal abnormalities | 119 | 0.8 |
| Diseases of the skin and subcutaneous tissue | 38 | 0.3 |
| Diseases of the blood and blood-forming organs and certain disorders involving the immune mechanism | 38 | 0.3 |
| Pregnancy, childbirth and puerperium | 29 | 0.2 |
| Diseases of the musculoskeletal system and connective tissue | 5 | 0.0 |
| **Total** | **14208** | **100** |
