# Supplementary material for: The economic impact of premature mortality in Cabo Verde: 2016–2020
Source: PLoS One. 2023 May 24;18(5):e0278590. doi: 10.1371/journal.pone.0278590 (PMC10208520; doi:10.1371/journal.pone.0278590)
Supplement: S3 Appendix — (DOCX) [file pone.0278590.s003.docx]

**Appendix 3: Rates of potential years of life lost by municipality, Cape Verde, 2016 to 2020**

| **YPLL and rates per year and county** | | | | | | | | | | | | | | | | |
| --- | --- | --- | --- | --- | --- | --- | --- | --- | --- | --- | --- | --- | --- | --- | --- | --- |
| **Municipality** | **Year** | | | | | | | | | | | | | | |  |
|  | **2016** | | | **2017** | | | **2018** | | | **2019** | | | **2020** | | |  |
|  | ***YPLL*** | ***TYPLL*** | ***TYPLLr*** | ***YPLL*** | ***TYPLL*** | ***TYPLLr*** | ***YPLL*** | ***TYPLL*** | ***TYPLLr*** | ***YPLL*** | ***TYPLL*** | ***TYPLLr*** | ***YPLL*** | ***TYPLL*** | ***TYPLLr*** |  |
| Ribeira Grande | 924 | 61.1 | 1.7 | 922 | 62.1 | 1.7 | 1086 | 74.4 | 2.1 | 1111 | 77.5 | 2.2 | 800 | 56.8 | 1.6 |  |
| Paul | 567 | 103.2 | 1.0 | 315 | 58.8 | 0.6 | 528 | 101.1 | 1.0 | 327 | 64.1 | 0.6 | 437 | 87.8 | 0.9 |  |
| Porto Novo | 861 | 53.7 | 1.7 | 437 | 27.4 | 0.8 | 927 | 58.5 | 1.8 | 903 | 57.3 | 1.8 | 1000 | 63.8 | 2.0 |  |
| São Vincent | 3891 | 50.5 | 7.8 | 3359 | 43.1 | 6.7 | 4847 | 61.6 | 9.6 | 4448 | 56.0 | 8.7 | 5292 | 66.1 | 10.2 |  |
| Ribeira Brava | 105 | 16.2 | 0.2 | 377 | 58.6 | 0.7 | 502 | 78.6 | 1.0 | 406 | 64.2 | 0.8 | 361 | 57.7 | 0.7 |  |
| Tarrafal de São Nicolau | 319 | 65.7 | 0.6 | 196 | 40.2 | 0.4 | 404 | 83.1 | 0.8 | 351 | 72.4 | 0.7 | 325 | 67.2 | 0.6 |  |
| Sal | 1805 | 53.1 | 4.0 | 1734 | 48.9 | 3.7 | 1796 | 48.6 | 3.6 | 1424 | 37.1 | 2.8 | 2113 | 53.1 | 4.0 |  |
| Boavista | 559 | 37.5 | 0.5 | 539 | 82.8 | 1.2 | 745 | 107.4 | 1.5 | 555 | 75.3 | 1.1 | 614 | 78.6 | 1.1 |  |
| Maio | 277 | 41.9 | 0.6 | 320 | 47.7 | 0.6 | 244 | 35.9 | 0.5 | 361 | 52.1 | 0.7 | 234 | 33.0 | 0.4 |  |
| Tarrafal | 1078 | 63.0 | 2.1 | 1179 | 69.0 | 2.3 | 992 | 58.2 | 1.9 | 1099 | 64.5 | 2.1 | 1232 | 72.4 | 2.4 |  |
| Santa Catarina | 2953 | 68.9 | 5.9 | 2205 | 51.0 | 4.4 | 2256 | 51.6 | 4.5 | 2690 | 61.0 | 5.3 | 2401 | 53.9 | 4.6 |  |
| Santa Cruz | 1583 | 63.7 | 3.1 | 1654 | 66.6 | 3.2 | 1410 | 57.0 | 2.7 | 1627 | 65.9 | 3.2 | 1651 | 67.0 | 3.2 |  |
| Praia | 9722 | 65.3 | 20.4 | 7721 | 50.6 | 15.8 | 9972 | 63.8 | 20.0 | 7748 | 48.4 | 15.1 | 9877 | 60.3 | 18.9 |  |
| São Domingos | 751 | 56.6 | 1.5 | 738 | 55.4 | 1.5 | 799 | 59.7 | 1.6 | 638 | 47.5 | 1.2 | 821 | 60.9 | 1.6 |  |
| São Miguel | 534 | 39.4 | 1.0 | 812 | 60.6 | 1.5 | 732 | 55.3 | 1.4 | 643 | 49.3 | 1.3 | 983 | 76.2 | 1.9 |  |
| São Salvador do Mundo | 313 | 39.1 | 0.6 | 284 | 35.4 | 0.6 | 463 | 57.8 | 0.9 | 395 | 49.3 | 0.8 | 420 | 52.3 | 0.8 |  |
| São Lourenço dos Órgãos | 453 | 68.9 | 0.9 | 404 | 61.7 | 0.8 | 260 | 40.0 | 0.5 | 206 | 31.7 | 0.4 | 334 | 51.7 | 0.7 |  |
| Ribeira Grande de Santiago | 223 | 28.0 | 0.4 | 356 | 44.5 | 0.7 | 556 | 69.4 | 1.1 | 365 | 45.3 | 0.7 | 519 | 64.1 | 1.0 |  |
| Mosteiros | 333 | 37.9 | 0.6 | 284 | 32.2 | 0.6 | 354 | 40.3 | 0.7 | 403 | 45.8 | 0.8 | 199 | 22.7 | 0.4 |  |
| São Felipe | 1142 | 57.6 | 2.2 | 1124 | 57.0 | 2.2 | 973 | 49.7 | 1.9 | 1196 | 61.5 | 2.3 | 1082 | 56.1 | 2.1 |  |
| Santa Catarina do Fogo | 183 | 36.8 | 0.4 | 204 | 41.0 | 0.4 | 312 | 62.7 | 0.6 | 256 | 51.5 | 0.5 | 482 | 97.4 | 0.9 |  |
| Brava | 279 | 53.0 | 0.5 | 246 | 47.0 | 0.5 | 572 | 110.6 | 1.1 | 384 | 74.9 | 0.7 | 279 | 55.0 | 0.6 |  |
| **Total** | **28851** | **57.4** | **57.4** | **25403** | **50.8** | **50.8** | **30727** | **60.8** | **60.8** | **27529** | **53.8** | **53.8** | **31452** | **60.8** | **60.8** |  |
