# Supplementary material for: The economic impact of premature mortality in Cabo Verde: 2016–2020
Source: PLoS One. 2023 May 24;18(5):e0278590. doi: 10.1371/journal.pone.0278590 (PMC10208520; doi:10.1371/journal.pone.0278590)
Supplement: S4 Appendix — (DOCX) [file pone.0278590.s004.docx]

**Appendix 4: Rates of years of potential productive life lost by municipality, Cape Verde, 2016 to 2020**

| **YPPLL and rates by year and municipalities** | | | | | | | | | | | | | | | |
| --- | --- | --- | --- | --- | --- | --- | --- | --- | --- | --- | --- | --- | --- | --- | --- |
| **Municipality** | **Year** | | | | | | | | | | | | | | |
|  | **2016** | | | **2017** | | | **2018** | | | **2019** | | | **2020** | | |
|  | ***YPPLL*** | ***TYPPLL*** | ***TYPPLLr*** | ***YPPLL*** | ***TYPPLL*** | ***TYPPLLr*** | ***YPPLL*** | ***TYPPLL*** | ***TYPPLLr*** | ***YPPLL*** | ***TYPPLL*** | ***TYPPLLr*** | ***YPPLL*** | ***TYPPLL*** | ***TYPPLLr*** |
| Ribeira Grande | 560 | 37.1 | 1.0 | 505 | 34.0 | 1.0 | 605 | 41.5 | 1.2 | 610 | 42.6 | 1.2 | 373 | 26.4 | 0.7 |
| Paul | 308 | 56.0 | 0.6 | 205 | 38.3 | 0.4 | 290 | 55.5 | 0.6 | 175 | 34.4 | 0.3 | 205 | 41.2 | 0.4 |
| Porto Novo | 510 | 31.8 | 1.0 | 238 | 14.9 | 0.5 | 570 | 36.0 | 1.1 | 580 | 36.8 | 1.1 | 573 | 36.5 | 1.1 |
| São Vincent | 2298 | 29.8 | 4.6 | 1755 | 22.5 | 3.5 | 2563 | 32.6 | 5.1 | 2323 | 29.3 | 4.5 | 2805 | 35.0 | 5.4 |
| Ribeira Brava | 50 | 7.7 | 0.1 | 150 | 23.3 | 0.3 | 323 | 50.6 | 0.6 | 240 | 38.0 | 0.5 | 170 | 27.2 | 0.3 |
| Tarrafal de São Nicolau | 210 | 43.2 | 0.4 | 63 | 12.9 | 0.1 | 213 | 43.8 | 0.4 | 175 | 36.2 | 0.3 | 195 | 40.4 | 0.4 |
| Sal | 1005 | 29.5 | 2.2 | 733 | 20.6 | 1.5 | 973 | 26.3 | 2.0 | 765 | 19.9 | 1.5 | 1115 | 28.0 | 2.1 |
| Boavista | 390 | 26.2 | 0.4 | 358 | 55.0 | 0.8 | 448 | 64.5 | 0.9 | 213 | 28.8 | 0.4 | 413 | 52.8 | 0.8 |
| Maio | 165 | 25.0 | 0.3 | 203 | 30.3 | 0.4 | 100 | 14.7 | 0.2 | 168 | 24.2 | 0.3 | 75 | 10.6 | 0.1 |
| Tarrafal | 558 | 32.6 | 1.1 | 613 | 35.8 | 1.2 | 535 | 31.4 | 1.0 | 603 | 35.4 | 1.2 | 728 | 42.8 | 1.4 |
| Santa Catarina | 1405 | 32.8 | 2.8 | 1305 | 30.2 | 2.6 | 1173 | 26.8 | 2.3 | 1413 | 32.0 | 2.8 | 1328 | 29.8 | 2.6 |
| Santa Cruz | 708 | 28.5 | 1.4 | 873 | 35.2 | 1.7 | 720 | 29.1 | 1.4 | 955 | 38.7 | 1.9 | 1008 | 40.9 | 2.0 |
| Praia | 5878 | 39.5 | 12.3 | 4330 | 28.4 | 8.9 | 5888 | 37.6 | 11.8 | 4733 | 29.6 | 9.3 | 5550 | 33.9 | 10.6 |
| São Domingos | 500 | 37.7 | 1.0 | 490 | 36.8 | 1.0 | 400 | 29.9 | 0.8 | 313 | 23.3 | 0.6 | 540 | 40.1 | 1.1 |
| São Miguel | 315 | 23.2 | 0.6 | 513 | 38.3 | 1.0 | 403 | 30.4 | 0.8 | 388 | 29.7 | 0.8 | 463 | 35.8 | 0.9 |
| São Salvador do Mundo | 210 | 26.3 | 0.4 | 183 | 22.8 | 0.4 | 233 | 29.1 | 0.5 | 153 | 19.0 | 0.3 | 248 | 30.9 | 0.5 |
| São Lourenço dos Órgãos | 250 | 38.0 | 0.5 | 283 | 43.2 | 0.5 | 155 | 23.8 | 0.3 | 85 | 13.1 | 0.2 | 200 | 31.0 | 0.4 |
| Ribeira Grande de Santiago | 148 | 18.5 | 0.3 | 153 | 19.1 | 0.3 | 315 | 39.3 | 0.6 | 198 | 24.6 | 0.4 | 248 | 30.6 | 0.5 |
| Mosteiros | 208 | 23.6 | 0.4 | 130 | 14.8 | 0.3 | 173 | 19.6 | 0.3 | 270 | 30.8 | 0.5 | 90 | 10.3 | 0.2 |
| São Felipe | 595 | 30.0 | 1.1 | 633 | 32.1 | 1.2 | 630 | 32.2 | 1.2 | 605 | 31.1 | 1.2 | 545 | 28.3 | 1.1 |
| Santa Catarina do Fogo | 118 | 23.7 | 0.2 | 35 | 7.0 | 0.1 | 140 | 28.2 | 0.3 | 140 | 28.2 | 0.3 | 185 | 37.4 | 0.4 |
| Brava | 168 | 31.8 | 0.3 | 150 | 28.7 | 0.3 | 268 | 51.7 | 0.5 | 168 | 32.7 | 0.3 | 165 | 32.6 | 0.3 |
| **Total** | **16553** | **32.9** | **33** | **13895** | **27.8** | **28** | **17113** | **33.8** | **34** | **15268** | **29.8** | **29.8** | **17218** | **33.3** | **33.3** |
