# Supplementary material for: The economic impact of premature mortality in Cabo Verde: 2016–2020
Source: PLoS One. 2023 May 24;18(5):e0278590. doi: 10.1371/journal.pone.0278590 (PMC10208520; doi:10.1371/journal.pone.0278590)
Supplement: Attachment — Submitted filename: Response to Reviewers.docx [file pone.0278590.s005.docx]

Dear Editor,

We would like to thank the editor and reviewers for their comments on our manuscript**-The economic impact of premature mortality in Cabo Verde: 2016 - 2020**

Following in italics are our responses to the reviewers’ comments.

.Reviewer #1: the paper in general was well written. I have some issues which I need the author to address.

1. I felt the terms YPPL and CPL should be defined not just YPLL to enable readers understand the concept of the article.

*Ans. Definations of YPPLL and CPL have been provided*

2. line 160, table 1, age ranges used is not conventional, I advise re-analysing using decades of life.

*Ans. Line 160 Table 1 have been corrected according to WHO standards.*

3. Why is the author lumping parasitic disease and certain infections ? Parasitic diseases are also infections.... what certain diseases does the author means in this study?

*Ans.We used the description used in International Statistical Classification of Diseases and Related Health Problems, 10th Revision to classify the groups of causes of death.*

[*https://icd.who.int/browse10/2016/en*](https://icd.who.int/browse10/2016/en)

*Certain infectious and parasitic diseases refering to diseases with codes A00 to B99*

4. The author should state what accounted for the remaining 18.3% of deaths among the study population on line 165.

*Ans. 18.3% was distributed among the rest of the causes of death in the table. We only mentioned the principal causes in our manuscript.*

5. Table 2, line 170, causes of deaths should be arranged using the variable with highest frequency and then in a decreasing fashion.

*Ans. Table 2, line 170 has been corrected as suggested*.

6. I have detected grammatical errors and incorrect use of tenses which should be addressed. Punctuation marks should be inserted appropriately for example on line 184, comma should be inserted after'. table'.

*Ans. Corrections were done as suggested.*

7. I felt the conclusion was not concise and has not summarised the findings from the study as such, the main cause of premature mortality should be stated clearly.

*Ans. We revised the conclusion as suggested*

Once again, our thanks to the editor and reviewers

Best regards

Ngibo Mubeta Fernandes
